# Fertility-Sparing Surgery for Ovarian Cancer

**DOI:** 10.3390/jcm10184235

**Published:** 2021-09-18

**Authors:** Geoffroy Canlorbe, Nathalie Chabbert-Buffet, Catherine Uzan

**Affiliations:** 1 Department of Gynecological and Breast Surgery and Oncology, Pitié-Salpêtrière, Assistance Publique des Hôpitaux de Paris (AP-HP), University Hospital, 75013 Paris, France; catherine.uzan@aphp.fr; 2 Centre de Recherche Saint-Antoine (CRSA), INSERM UMR_S_938, Cancer Biology and Therapeutics, Sorbonne University, 75012 Paris, France; nathalie.chabbert-buffet@aphp.fr; 3 University Institute of Cancer, Sorbonne University, 75013 Paris, France; 4 Department of Gynaecology, Obstetrics and Reproductive Medicine, Tenon University Hospital, Assistance Publique des Hôpitaux de Paris (AP-HP), Sorbonne University, 75020 Paris, France

**Keywords:** ovarian cancer, fertility sparing surgery, conservative surgery, borderline ovarian, epithelial ovarian cancer

## Abstract

(1) Background: although most patients with epithelial ovarian cancer (EOC) undergo radical surgery, patients with early-stage disease, borderline ovarian tumor (BOT) or a non-epithelial tumor could be offered fertility-sparing surgery (FSS) depending on histologic subtypes and prognostic factors. (2) Methods: we conducted a systematic review to assess the safety and fertility outcomes of FSS in the treatment of ovarian cancer. We queried the MEDLINE, PubMed, Cochrane Library, and Cochrane (“Cochrane Reviews”) databases for articles published in English or French between 1985 and 15 January 2021. (3) Results: for patients with BOT, FSS should be offered to young women with a desire to conceive, even if peritoneal implants are discovered at the time of initial surgery. Women with mucinous BOT should undergo initial unilateral salpingo-oophorectomy, whereas cystectomy is an acceptable option for women with serous BOT. Assisted reproductive technology (ART) can be initiated in patients with stage I BOT if infertility persists after surgery. For patients with EOC, FSS should only be considered after staging for women with stage IA grade 1 (and probably 2, or low-grade in the current classification) serous, mucinous or endometrioid tumors. FSS could also be offered to patients with stage IC grade 1 (or low-grade) disease. For women with serous, mucinous or endometrioid high-grade stage IA or low-grade stage IC1 or IC2 EOC, bilateral salpingo-oophorectomy and uterine conservation could be offered to allow pregnancy by egg donation. Finally, FSS has a large role to play in patients with non- epithelial ovarian cancer, and particularly women with malignant ovarian germ cell tumors.

## 1. Introduction

The aim of conservative and functional surgery in an oncology setting is to preserve an organ’s functionality and to avoid radical resection when possible. This approach is increasingly used in oncologic gynecologic surgery where fertility-sparing surgery (FSS) aims to preserve the ovarian tissue and the uterus. Moreover, FSS can improve sexual function and the psychological wellbeing of patients, both of which are negatively impacted after cancer diagnosis and treatment [1]. Cryopreservation may also be an option prior to surgery if the risk of gonadal damage is high [2].

Ovarian cancers are classified into epithelial (including borderline ovarian tumors (BOT) and malignant ovarian tumors) and non-epithelial cancer. Although most patients with epithelial ovarian cancer will undergo radical surgery -the gold standard-patients with early-stage disease, BOT, or a non-epithelial tumor could be offered FSS depending on histologic subtypes and prognostic factors [3,4].

The aim of this systematic review is to assess safety and fertility outcomes of FSS in the treatment of ovarian cancer.

## 2. Materials and Methods

The MEDLINE, PubMed, Cochrane Library, and Cochrane (“Cochrane Reviews”) databases were queried with the following terms: ovarian cancer, fertility sparing surgery, conservative surgery, borderline ovarian, epithelial ovarian cancer, mucinous ovarian cancer, serous ovarian cancer, non-epithelial ovarian cancer, germ-cell tumor, sex cord stromal tumor, dysgerminomas, endodermal sinus tumor, malignant teratoma, granulosa cell tumor, Sertoli-Leydig cell tumor, and theca cell tumor. The database search was further supplemented with original articles, reviews, and meta analyses, including the studies cited therein. Only articles published in English or French between 1985 and 15 January 2021 were included.

## 3. Borderline Ovarian Tumors

### 3.1. Modalities of Fertility-Sparing Surgery and Clinical Outcomes

BOT predominantly affects younger women of childbearing age for whom fertility is a major issue. However, the current standard treatment of BOT consists of total abdominal hysterectomy and bilateral salpingo-oophorectomy, peritoneal cytology, omentectomy, and multiple peritoneal biopsies. Adjuvant therapy is not usually necessary unless invasive peritoneal implants are detected [5]. While the prognosis of BOT is excellent, late recurrences (after 5 or 10 years) can occur [6]. FSS in patients with BOT consists of preserving the uterus and at least part of one ovary. In 2013, Daraï et al. conducted a review to analyze the outcomes of FSS (salpingo-oophorectomy or cystectomy) in patients with BOT [7]. They concluded that the risk of relapse was higher after FSS compared with standard treatment, with a global recurrence risk estimated at 13% (95% Confidence Interval (CI) 10–16%). The recurrence rate was correlated with the type of FSS performed with a higher risk (between 10 and 42%) observed in patients undergoing cystectomy [7]. Nevertheless, some authors report similar recurrence rates for cystectomy and salpingo-oophorectomy. Palomba et al. [8,9] conducted a randomized trial in 32 patients who underwent laparoscopy for bilateral serous BOT. The patients were randomized into two groups: bilateral cystectomy or unilateral salpingo-oophorectomy on the largest lesion and contralateral cystectomy. They found no difference between the procedures in terms of the cumulative recurrence rates with a follow-up of 81 months. However, although the cumulative pregnancy rate and cumulative probability of a first pregnancy were higher in the group of patients treated with bilateral cystectomy [8], time to first recurrence was shorter and the rate of radical treatment of the recurrence was higher in this group [9]. This study therefore suggests that patients with bilateral serous BOT who wish to conceive should undergo bilateral cystectomy if technically feasible.

The optimal FSS treatment for women with BOT would thus be unilateral adnexectomy, which is associated with a lower risk of relapse. Women with bilateral serous tumors and/or with only one ovary (previous history of adnexectomy) can undergo initial cystectomy and, in case of BOT relapse on the remaining ovary, another cystectomy could be performed to preserve fertility. In this case, a complete preoperative workup should be performed, including:

(1) Magnetic resonance imaging (MRI) to assess possible healthy functional ovarian tissue.
(2) An oncofertility consultation to discuss whether a preoperative fertility preservation technique could be performed.

### 3.2. Survival of Patients after Fertility-Sparing Surgery

We have seen that FSS is associated with a higher recurrence rate compared to radical treatment but it does not affect survival rates because most recurrences are also borderline and can be managed by further surgery. Bendifallah et al. developed a nomogram to predict recurrence in patients with early- and advanced-stage mucinous and serous BOT. An increased risk of recurrence was associated with the surgical procedure (radical vs. FSS); International Federation of Gynecology and Obstetrics (FIGO) stage; age at diagnosis; histologic subtype; and completeness of surgery [10].

Nevertheless, a major challenge is to identify women at risk of invasive recurrence, which can be lethal. In the literature, 47 cases of women with BOT progressing to invasive carcinoma have been described with an estimated risk of progression at around 2–3% [11]. Twenty of these invasive recurrences were observed in patients with serous BOT (mean time to progression: 75 months (range, 11–310)), 24 with mucinous BOT (mean time to progression: 33 months (range, 5–82)), and in three patients of unknown histologic subtype. Recurrences associated with mucinous BOT, though less frequent, were more likely to be invasive. In a recent analysis of 212 patients with advanced stages of BOT, the largest series of patients to date [12], of the 38 (58%) patients who underwent FSS and who experienced recurrence, eight had invasive disease (three patients died). Compared with radical surgery, the use of FSS was a prognostic factor for disease-free survival (*p* < 0.0001) but did not affect overall survival, as mentioned above.

A micropapillary pattern and mucinous subtype were found to be associated with a higher rate of progression to invasive disease after FSS in a large series of women with stage I BOT [13]. A recent study by Jia et al. compared FSS (either bilateral cystectomy or unilateral oophorectomy/cystectomy) and radical surgery in patients with bilateral serous BOT who self-selected one of the three treatment groups after preoperative counseling. The authors reported that a preoperative cancer antigen (CA)-125 > 300 U/mL, fertility preservation, and micropapillary pattern were independently associated with a poor disease-free survival on multivariate analysis (*p* = 0.001, 0.03 and 0.026, respectively) [14]. Fourteen patients (15%) experienced invasive recurrence which was significantly associated with a micropapillary pattern (*p* = 0.006) [14]. Nevertheless, it is important to point out that only 41 patients (43.6%) in this series received complete staging during their initial surgery, without a second look, implying that the FIGO stage and number of patients with extraovarian implants might have been underestimated.

The risk of recurrence is lower in patients with mucinous BOT but, when recurrence occurs, it tends to be more invasive. Among the 47 patients with BOT who underwent a cystectomy and developed invasive disease, five of the 11 patients with serous BOT were alive and disease-free at the end of follow-up compared with one of the nine patients with mucinous BOT [11]. There was a higher rate of extra-abdominal metastases as the first recurrence site in the patients with mucinous BOT (four pleural, lung or bone metastases). None of the first recurrence sites were extra-abdominal in the patients with invasive serous BOT [11]. These results suggest that women with mucinous BOT should undergo initial unilateral salpingo-oophorectomy, whereas cystectomy is an acceptable option for women with serous BOT, which has a lower risk of lethal recurrence, in the absence of other high-risk factors.

In a large German series of 950 patients, two-thirds of whom had serous BOT (*n* = 694) and just under one-third mucinous BOT (*n* = 290), 2.3% of the lesions transformed into histologically proven invasive disease. In this series, five-year progression-free and overall survival rates were 12% and 50%, respectively [15].

### 3.3. Fertility Results after Fertility-Sparing Surgery

The observed pregnancy rates after FSS for patients with BOT and a desire for pregnancy lie between 32 and 88% (Table 1) [12,14,16,17,18,19,20,21,22,23,24,25,26,27]. Three main factors impact fertility rates: the type of FSS performed, the patient’s age, and the histologic subtype of the tumor.

As mentioned above, both the cumulative pregnancy rate and cumulative probability of a first pregnancy were found to be higher after cystectomy compared with salpingo-oophorectomy and collateral cystectomy [8,9]. As already mentioned above, cystectomy should therefore be the treatment of choice for women with serous BOT who wish to become pregnant.

Secondly, the chances of becoming pregnant decrease with age. Fauvet et al. [17] showed that spontaneous fertility outcomes were poorer in women over 40 years of age. Similarly, Kanat-Pektas et al. [19] reported that the median age of patients who conceived was lower than that of patients who could not (36 vs. 45 years). Nevertheless, it is important to take into account that neither of these studies provided sufficient data about ovarian reserve (antral follicle count or serum anti-Müllerian hormone (AMH) levels). In the large German series, Trillsch et al. reported that the younger patients of child-bearing age with relapse were at a higher risk of disease recurrence despite favorable survival outcomes [28]. In a longitudinal study from the French National Cancer Network [26] reporting 52 patients aged 18 to 42 years who underwent FSS for BOT and wished to become pregnant, two-thirds of the patients had a live birth after FSS. However, both recurrence and live-birth rates were independent of age, and the authors did not identify a specific age as a cut-off for risk of recurrence, or a time from surgery after which more radical surgery should be undertaken to reduce the risk of recurrence. As almost a quarter of the live births occurred after recurrence, with no further events up to the end of the study period, these results are in line with the recommendation to perform a second FSS after recurrence of a local BOT for women who desire to become pregnant.

Thirdly, in a study investigating whether fertility outcome was related to age, tumor histology or type of surgery, Kanat-Pektas et al. [19] noted that fertility results were better in patients with non-serous (mainly mucinous) BOT: 87% of the women conceived in the non-serous BOT group compared with 13% in the group composed mainly of women with serous BOT. This can be explained by the fact that patients with serous BOT are more likely to have bilateral tumors, peritoneal disease or a previous history of infertility which can affect subsequent fertility [29]. Higher pregnancy rates were reported in an Asian series where women with mucinous tumors are more likely to be treated with FSS than elsewhere.

Finally, a laparoscopic approach and a 2- or 3-step surgical procedure (initial, restaging, second look) may also affect fertility rates, although there are no specific data to support this. Further studies are thus merited. A nomogram designed to predict the live-birth rate after FSS for patients with BOT includes FIGO stage, age at diagnosis, histologic subtype, and type of surgery [30].

In a meta-analysis, Jiao et al. reported that the risk of recurrence was significantly higher in patients with unilateral cystectomy (odds ratio (OR), 2.49; 95% CI, 1.86–3.33) or serous BOT (OR, 3.15; 95% CI, 1.97–5.02). The surgical approach, i.e., laparoscopy or laparotomy, did not affect the risk of recurrence (OR, 0.96; 95% CI, 0.57–1.60) [31].

Some patients with BOT who have FSS will experience infertility. In this case, the following question arises: as some studies suggest that infertility treatment, and more specifically clomiphene citrate, are implicated in the genesis of gynecologic cancers, can these women be offered assisted reproductive technology (ART)? However, in vitro fertilization (IVF) procedures have not been associated with an increased risk of BOT or ovarian cancer rates [32]. Furthermore, in vitro data suggest that neither gonadotropins nor high doses of estrogen induce proliferation of borderline cell cultures [33].

Therefore, the answer to the question is that ART is an option for women with BOT-associated infertility even though only a few authors have reported their experiences [9,17,20,34,35,36,37,38,39,40,41,42,43,44,45,46,47,48].

**Figure 1:**
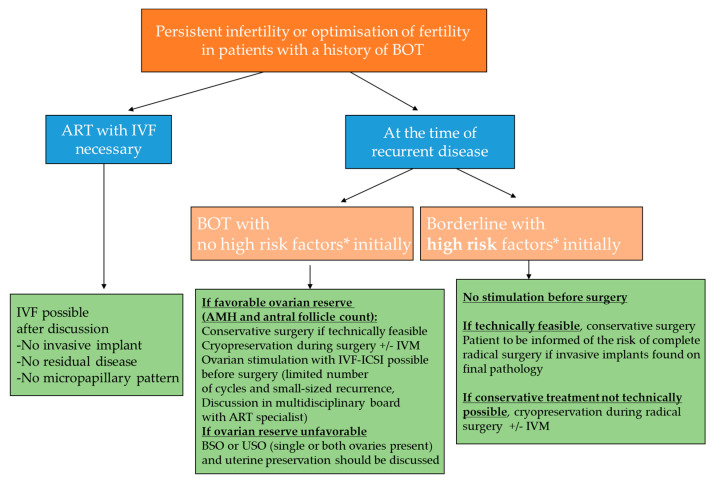
Management of infertility (or optimization of infertility) in a young patient with previous history of BOT (ART, assisted reproductive technology; IVF, in vitro fertilization; ICSI, intracytoplasmic sperm injection; IVM, in vitro maturation; BSO, bilateral salpingo-oophorectomy; USO, unilateral salpingo-oophorectomy; AMH: anti-Mullerian hormone; * High-risk recurrent borderline tumor: defined by a recurrent case presenting radiologic (on magnetic resonance imaging) or clinical (recurrence associated with implants) suspicious lesion at the time of the recurrence or histologic high risk factors (peritoneal implants, mucinous tumor with intraepithelial carcinoma, micropapillary patterns, stromal microinvasion) during the treatment of the first tumor (ref Darai et al. Human reprod 2013).

Most of these studies concern IVF rather than simple ovarian stimulation. A literature review of these series [7] found a pooled estimate for pregnancy of 80% (95% CI: 68–92%), and a pooled estimate for recurrence of 23% (95% CI: 6–39%). This low rate of recurrence is probably because women who are eligible for ART tend to have a better prognosis and early-stage BOT. Overall, women with BOT who wish to conceive should be followed in a specialized center with associating oncologists and fertility experts who can assess the feasibility of FSS on an individual basis along with alternative options to preserve fertility, including ART. Figure 1 summarizes the various options in this setting.

Finally, it is worth focusing on BOT in children and adolescents. A recent literature review identified 300 cases of BOT occurring in children and young women aged between 3 and 25 years (15 of whom were premenarchal) [49]. The largest series was a SEER (Surveillance, Epidemiology and End Results) Database study from Nasioudis et al. with 114 patients [50] which found that serous histology was more common than mucinous in this age group (53.5% vs. 44.8%). However, while pediatric BOTs are rare and difficult to predict preoperatively, prognosis is excellent after FSS even in patients with advanced stages. FSS should therefore be the gold standard of treatment in children and adolescents.

## 4. Epithelial Ovarian Cancer

### 4.1. Indications of Fertility-Sparing Surgery

As for BOT, the standard surgical procedure for women with EOC is radical: hysterectomy with bilateral salpingo-oophorectomy. It is somewhat difficult to analyze the outcome of FSS in this setting because many of the reported series either mix epithelial and non-epithelial ovarian cancers, or include invasive cancers and BOT, considering them as epithelial. Very few series report FSS outcomes in EOC exclusively.

Table 2 provides a summary of reported recurrence risk and survival rates after FSS for patients with EOC [51,52,53,54,55,56,57,58,59,60,61,62,63,64,65,66,67,68,69,70,71,72,73,74,75,76,77,78,79,80,81].

P DiSaia was the first to offer FSS for EOC in highly selected patients, i.e.: with well-encapsulated stage IA EOC without peritumoral adhesions, ovarian capsule lymphatic channels and/or mesovarium invasion, and negative peritoneal washings; with a desire to conceive; and a willingness to undergo close gynecologic follow-up [83].

The first series specifically describing FSS in women with EOC was published by Colombo et al. [82] in 1994 and by Zanetta et al. [51] in 1997. Together they included 56 selected patients (i.e., stage IA to IC disease, any grade) who underwent FSS. Survival was excellent: for example, Colombo et al. reported a five-year survival rate of 96%.

An American multicenter study comprising 52 patients with early-stage EOC who underwent FSS [52] reported estimated five-year and 10-year overall survival rates of 98% and 93%, respectively. The authors went on to suggest that FSS could be performed in stage I EOC of any grade. 

In 2005, a French multicenter retrospective study described a series of 34 patients who had undergone FSS for EOC [53] with a systematic review of slides, complete staging surgery and chemotherapy for patients with stage ≥IC. They reported one case of recurrence in the 13 patients with stage I grade 1 disease, and eight cases of recurrence in 20 patients with grade 2/3 stage IA to IC disease. They concluded that FSS should be reserved for patients with a FIGO stage ≤ IA.

A study by Park et al. in 2008 [55], including 62 patients with EOC, 59 of whom had early-stage disease, found that patients with stage IC or grade 3 tumors had significantly poorer survival (5-year survival: 88%). The authors concluded that FSS was an option in young patients with EOC of stages IA–C, grades 1–2.

A large Japanese multicenter study [60] of 211 patients with EOC who underwent FSS in 30 institutions reported five-year recurrence-free survival rates of 97.8% for stage IA with favorable histology (grade 1, grade 2, not clear-cell), 100% for stage IA clear-cell, 33.3% for stage IA grade 3, 92.1% for stage IC with favorable histology, 66% for stage IC clear-cell, and 66.7% for stage IC grade 3. The authors suggested that FSS was an option in patients with stage IA disease either with a favorable histologic subtype or clear-cell histology, or in patients with stage IC with a favorable histology only. However, they specified that FSS should be avoided in patients with grade 3 tumors.

A large analysis of FSS with preservation of the ovary in stage IA or IC disease in the SEER database confirmed that FSS did not affect survival rates [59]. Nevertheless, the authors pointed out that “to detect a 20% difference in survival for patients with stage IC disease, a cohort of 1282 patients with 52 deaths is required”. Therefore, it is not possible to make firm conclusions about the safety of FSS in patients with stage IC disease since the published series to date are all underpowered.

Another large retrospective study was published by Fruscio et al. in 2013 and updated in 2016 [65,66]. They found that the oncologic prognosis was the same for the 240 patients treated with FSS as for the patients who underwent radical treatment. Prognosis was worse in women with grade 3 but independent of the type of surgery. The authors conclude that FSS can be offered to all young patients when the tumor is limited to the ovaries. Patients with grade 3 tumors are more likely to experience distant recurrences and these patients should be closely monitored. 

These data have been confirmed by more recent studies. In 2017, Jiang et al. published a retrospective study of 108 patients of reproductive age (≤40 years) diagnosed with stage I EOC who were treated either with FSS (48.1%) or radical surgery (51.9%). After a median follow-up of 83 months, multivariate analysis revealed that the only independent risk factors for disease-free survival and tumor-specific survival were grade 3 or clear-cell carcinoma. The authors thus concluded that FSS was safe for patients of reproductive age with grade 1–2, stage I EOC [73].

The largest study was published by Melamed et al. in 2017 [74]. This American cohort study, using the National Cancer Database, identified 1726 women with stage IA and unilateral IC EOC of whom 825 (47.8%) underwent FSS. FSS was not associated with hazard of death (hazard ratio 0.80, 95% CI 0.49–1.29, *p* = *0*.36). Of particular interest, they observed that in patients with high-risk features such as a clear-cell histology, grade 3, or stage IC, 10-year survival was 80.5% (95% CI 68.5–88.3) among women who underwent FSS and 83.4% (95% CI 76.0–88.7) among those who had conventional surgery (hazard ratio 0.86, 95% CI 0.49–1.53, *p* = 5.61). The authors concluded that compared with conventional surgery, FSS was not associated with an increased risk of death in young women with stage I EOC. Nonetheless, given the limited number of patients with clear-cell and other high-grade histology included in the study, the authors recommended that preoperative counseling should warn women that the safety of FSS is less certain in patients with clear-cell tumors or other high-grade histology [74]. In 2017, Gouy et al. published a retrospective survey concerning 21 patients with unilateral mucinous ovarian cancer (mOC) who underwent unilateral salpingo-oophorectomy and who, with one exception, completed peritoneal staging surgery [72]. After a median time of follow-up of 46 months (range, 1–179), recurrence was observed in two patients: one who had an expansile-type tumor and the other an infiltrative type. Based on these results, the authors suggest that the type of mOC (i.e., infiltrative or expansile) does not impact the oncologic outcomes in stage I mOC, and that FSS could be offered for women with early-stage infiltrative-type tumors using the same criteria as for expansile-type tumors.

Similarly, Hanatani et al. presented the results of a retrospective study including 583 patients (325 patients with cancer and 258 with BOT) <40 years old. In this study, multivariate analysis revealed that the independent prognostic factors for overall survival were age, stage, histology, and ascitic fluid cytology, but not FSS [76].

More recently, in 2020, Bogani et al. investigated the 10-year outcomes in a large series of women with apparent early-stage ovarian cancer who underwent either FSS (*n* = 34) or radical surgery (*n* = 148). Their results showed that the type of surgery (FSS vs. radical) did not affect survival in the high-risk group (≥grade 3 or ≥stage IC) [78].

Finally, a meta analysis by Liu et al. [79] including eight studies demonstrated that the type of surgery did not seem to affect overall or disease-free survival for patients with stage 1 EOC. Neither did disease stage, tumor grade or histology appear to influence outcomes [79].

Watanabe et al. published a retrospective study including 29 EOC patients (stage IA, *n* = 14; stage IC1 *n* = 6; stage IC3, *n* = 9) aged ≤ 40 years who had undergone FSS [80]. The respective 5-year relapse-free and overall survival rates were 90.9% and 100%, respectively, for stage IA/IC1, and 43.8% and 87.5% for stage IC3. Significant differences in relapse-free survival were observed between patients with stage IA/IC1 and IC3 (*p* = 0.026). However, there was no significant difference in overall survival between patients with 1A/1C1 and those with 1C3 (*p* = 0.712). According to the authors, these results confirm that FSS may be an acceptable treatment method for stage IA and IC1 EOC, exhibiting a favorable reproductive outcome. However, the safety of FSS for treating stage IC3 EOC is uncertain and warrants further investigation [80].

While prognosis remains poor for women who experience recurrence outside the preserved ovary after FSS [84], prognosis is good if the recurrence is limited to the preserved ovary [85].

Patients with a “borderline” indication for FSS (i.e., stage IA grade 3 disease, stage IB or IC grade 2 or 3 disease) could undergo removal of both ovaries and preservation of the uterus (including no uterine curettage for staging), and subsequently attempt pregnancy using egg donation. This approach has not been explored to date in EOC but deserves to be evaluated. In the SEER database analysis, uterine preservation was not found to impact survival in women with stage IA or IC disease [59].

In light of recent data [86,87], the best treatment option for patients with a clear-cell stage I tumor should be discussed by dedicated tumor boards on a case-by-case basis. In France, for example, a national expert website has been developed (www.ovaire-rare.org), in which cases of rare ovarian tumors and FSS for EOC can be discussed.

FSS is obviously not performed in women with disease extending beyond the ovaries because of the high risk of recurrence [53,55]. Nevertheless, some cases have been reported in the literature and were analyzed in the review by Petrillo et al. [87]. The authors report 21 patients with stage II–III disease who underwent FSS. Nine of the patients (42.8%) experienced recurrence and five (23.8%) died of the disease. Radical surgery thus remains the standard treatment for advanced EOC.

FSS generally consists of the conservation of at least the contralateral ovary and the uterus with a staging surgery. However, information about the clinical outcome in women who undergo a cystectomy as a fertility-preserving option is lacking. Recently, Kajiyama et al. [77] presented the results of a retrospective study including eight patients with early-stage EOC treated with cystectomy as FSS. Two of the patients (IA, endometrioid histology, and IC3 mucinous histology) experienced recurrence in the pelvic cavity and bilateral ovaries, respectively, and one died of the disease. The authors concluded that more research is required to clarify the applicability of cystectomy for FSS in this setting.

### 4.2. Fertility Results

Table 3 summarizes the fertility results found in the literature.

Only 12 series have reported fertility data after EOC [52,55,57,58,60,64,65,66,67,72,73,75,77]: of the 42% of patients who wished to be pregnant after FSS (339/802), 67% achieved pregnancy (226/339), resulting in 264 live births.

Ovarian stimulation or IVF remain contraindicated for women with persistent infertility.

Patient follow-up is based on clinical examination, blood markers, and systematic imaging (abdomino-pelvic ultrasonography).

The interest of completion of surgery after childbearing age (or after 40 years in patients who have not been pregnant) is still under discussion. Nevertheless, to reduce the risk of recurrence, removal of the remaining ovary should be considered in women who no longer intend to conceive, integrating information about BRCA mutations.

## 5. Non-Epithelial Ovarian Cancer

Compared to EOC, non-epithelial malignant tumors are characterized by: 1. disease onset at an early age, and 2. an overall good prognosis (even in case of extra-ovarian disease) due to excellent response to chemotherapy. The tumors can be classified into two main groups: malignant ovarian germ cell tumors (MOGCT) and sex cord stromal tumors (SCST).

### 5.1. Malignant Ovarian Germ Cell Tumors

Most studies exploring the results of FSS in non-epithelial cancers concern MOGCTs (Table 4) [75,88,89,90,91,92,93,94,95,96,97,98,99,100,101,102,103,104,105,106,107,108,109].

The most frequent MGCTs are dysgerminomas, endodermal sinus tumors (EST), malignant teratoma, or mixed subtypes. The standard chemotherapy regimen for such tumors is a combination of bleomycin, etoposide and cisplatin (the BEP regimen). Younger patients are usually managed by FSS after discussion about staging procedures (nodal or peritoneal). Biopsy of the contralateral ovary is not recommended in patients with non-dysgerminoma tumors and a macroscopically normal ovary. The situation is less clear for women with a dysgerminoma because of the risk of occult disease, which is observed in 10% of patients. For example, Boran et al. observed that two (11%) of the 17 patients with macroscopically normal contralateral ovary had occult involvement [97].

Table 4 summarizes the fertility results of series reported after 1995. Most young patients treated by the BEP regimen continue to menstruate and maintain endocrine ovarian function. In the largest series from Satoh et al. [102], all patients who underwent FSS recovered their menstrual cycle. Sixteen of 23 patients receiving BEP (70.0%) and 13 of 17 patients receiving non-BEP (76.5%) who were nulliparous at FSS and married at the time of investigation (although these are debatable criteria) gave birth to 21 and 19 healthy children, respectively. More recently, Park et al. [105] investigated 171 patients with early (*n* = 125) and advanced (*n* = 46) MOGCTs who underwent FSS, and reported a 5-year disease-free survival rate of 86%, and a 5-year overall survival rate of 97%. The 5-year disease-free survival was 84% for stage I and 89% for stage II–IV. The 5-year overall survival was 99% for stage I and 91% for stage II–IV. Yolk sac tumor, incomplete surgical staging, and residual tumor were independent prognostic factors. Interestingly, reproductive and obstetric outcomes were evaluable in 124 patients of whom 106 (85.5%) had regular menstruation, 12 (9.7%) had irregular menstruation, and six (4.8%) had premature menopause. Of the 20 patients who tried to conceive, 15 patients (75%) were successful, resulting in 21 pregnancies, and 13 (65%) gave birth to 20 healthy babies. Furthermore, a study by Turkmen et al. [110] concerning 69 patients with stage I and II MOGCTs (56 undergoing FSS) found that surgery type (FSS vs. radical surgery) and lymphadenectomy (performed vs. not performed) did not affect recurrence rates (*p* = 0.758, *p* = 0.271). However, recurrence was linked to surgical outcome (maximal vs. optimal and suboptimal) and type of tumor (dysgerminoma vs. nondysgerminoma) (*p* = 0.001, *p* = 0.021, respectively). Such conservative management of a part of one ovary could be considered for patients with bilateral involvement of both ovaries (as in the case of teratomas) or for patients with peritoneal disease treated by adjuvant chemotherapy (particularly in for dysgerminomas or malignant teratoma).

Completion surgery after childbearing is not necessary in these patients because of the high curability rates.

### 5.2. Sex Cord Stromal Tumors

The most frequent subtypes of SCSTs are granulosa cell, Sertoli-Leydig cell, and thecal cell tumors. Publications of FSS outcomes in women with SCSTs are scarce and mainly consist of case reports or short series [111,112,113,114,115,116,117,118,119,120,121,122].

In 2007, Zhang et al. published an analysis of the SEER database including 376 women treated for SCST: 71 young patients were treated using uterine preservation for stage I disease. The survival of patients treated by FSS and radical surgery was similar [117].

Two important characteristics of granulosa cell tumors affect FSS: bilaterality is only observed in between 2% and 8% of cases [118,119,120,121,122]; and these tumors are often associated with endometrial disorders (hyperplasia or cancers). Therefore, while random biopsies of the contralateral ovary (if macroscopically normal) are not necessary, uterine curettage should be systematically performed. The overall prognosis of granulosa cell tumors is good in early-stage disease (stage IA) and FSS is an option for young patients. However, prognosis is more reserved for women with higher stages (or in the case of ovarian rupture during the initial surgery), and FSS is not recommended in this setting. In 2018, Wang et al. [120] published a retrospective analysis of 113 patients with stage I ovarian granulosa tumor: 61 had FSS and 52 underwent radical surgery. After a median follow-up of 99.2 months (range 20.2–394.3 months), 30 patients had recurrent disease (17 in the FSS group and 13 in the radical surgery group). Multivariate analysis showed no difference in disease-free survival between the FSS and radical surgery groups (*p* = 0.550). In patients who underwent FSS, incomplete staging was significantly associated with the risk of recurrence (*p* = 0.024). Of the 22 patients who wished to conceive, 19 achieved 20 singleton pregnancies. The pregnancy rate was 86.4% and the live-birth rate was 95%. In 2019, Bergamini et al. [121] presented the results of a study comparing oncologic outcomes of FSS and radical surgery in 239 patients with apparent stage I adult-type granulosa cell tumors of the ovary treated within the MITO group (Multicenter Italian Trials in Ovarian cancer). Among the 78 patients (32.6%) in the FSS group, 49 (62.8%) underwent unilateral salpingo ovariectomy, 13 (16.7%) cystectomy, and 16 (20.5%) cystectomy followed by unilateral salpingo-oophorectomy. After a median follow up of 84 months, median disease-free survival was significantly worse in the FSS group (10-year disease-free survival was 50% in the FSS group vs. 74% in the radical surgery group, *p* = 0.006). No significant difference was detected between radical surgery and unilateral salpingo-oophorectomy (10-year disease-free survival 75% vs. 70%, respectively, *p* = 0.5). Disease-free survival was significantly worse in the cystectomy group compared with the unilateral salpingo-oophorectomy group (10-year disease-free survival 16% vs. 70%, respectively, *p* = 0.001). Patients undergoing cystectomy and subsequent unilateral salpingo-oophorectomy showed a better prognosis, even though significantly worse compared with unilateral salpingo-oophorectomy (10-year disease-free survival 41% vs. 70%, *p* = 0.05). On multivariate analysis, FIGO stage IC and cystectomy had a significant predictive value for worse survival. 

In 2019, Gouy et al. published a retrospective pathologic analysis by two expert pathologists of 23 patients with Sertoli-Leydig Cell Tumors (SLCT), and a literature review [112]. The results suggested that FSS should be performed for stage IA disease in children and in women of reproductive age. The difficulty in managing stage IA is determining whether to use an adjuvant treatment. In this population the risk of recurrence was around 7.5% (27/362), irrespective of the type of surgery (21/265-8% in the FSS group and 6/97-6% in the radical surgery group), but the risk of death after recurrence was as high as 70% (19/27). The same year Xu et al. published a series of seven patients with stage I SLCT of the ovary. Five patients underwent FSS (2IA, 3IC). All the patients were free of disease with a median follow-up of 13.5 months, the longest being 24 months (113). More recently, Durmus et al. (114) published a series of 17 patients with SLCT (13 IA, 2 IC1, and 2 IC2). Eight of the patients underwent total abdominal hysterectomy and bilateral salpingo-oophorectomy, seven underwent unilateral salpingo-oophorectomy or oophorectomy, and two underwent cystectomy with or without additional surgical staging procedures. Four patients received adjuvant chemotherapy. All 17 patients were alive and free of disease for 1–287 months after the diagnosis. Median follow-up time was 78 months. Besides the FIGO stage, poor prognosis for SLCT is correlated with poor tumor differentiation and the presence of heterologous elements, as specified in the 2012 European Society for Medical Oncology (ESMO) guidelines [123]. The guidelines recommend that adjuvant chemotherapy be considered for patients with stage I disease (without distinguishing between stages IA and IC) in the presence of these two factors. Schneider et al. showed that the presence of a retiform pattern was also an indicator of poor prognosis [124]. In 2014, the Study Group on Pediatric Rare Tumors described a series of 44 young patients with pediatric SLCT (median age 13 years) and confirmed that the differentiation grade, heterologous elements, and a retiform pattern were prognostic factors [125].

Extreme caution is required to avoid rupture when operating on young patients with a suspected ovarian mass (i.e., oophorectomy should be performed rather than cystectomy) especially if hyperandrogenism is suspected. Young et al. identified rupture as a factor of poor prognosis: the risk of recurrence increased from 30 to 38% for women with stage IC disease undergoing FSS, and from 14 to 36% for women undergoing radical surgery [126]. This poor prognosis associated with stage IC disease could be related to the preservation of the ovary (which raises the question of the safety of FSS), to the natural history of SLCT, or to both.

The prognosis of advanced stage disease (i.e., ≥stage II) is poor; advanced stages are associated with a high rate of death. In the literature review by Gouy et al. [112], 14 of the 19 patients with advanced stage disease experienced a recurrence, and 11 died.

In conclusion, for women of reproductive age with stage IA disease, FSS is safe and effective for treating ovarian SLCT. However, more data are needed to define the role of FSS to treat women with stage IC1.

The use of completion surgery after childbearing remains debatable in SCST [127].

## 6. Conclusions

For patients with BOT, FSS gives good fertility results and does not affect survival. It should therefore be offered to young women with a desire to conceive, even if peritoneal implants are discovered at the time of initial surgery. In the case of persistent infertility, ART can be initiated in patients with stage I BOT although the number of stimulation cycles should be limited.

In patients with EOC, FSS should only be considered in adequately staged patients, with stage IA grade 1 (and probably 2, or low-grade in the current classification) serous, mucinous or endometrioid tumors, and with close gynecologic follow-up. FSS could also be offered to patients with stage IC grade 1 (or low-grade) disease.

For women with serous, mucinous or endometrioid high-grade FIGO stage IA or low-grade FIGO stage IC1 or IC2 EOC tumors, bilateral salpingo-oophorectomy and uterine conservation could be offered to allow pregnancy by egg donation.

Finally, FSS has a large role to play in patients with non-epithelial ovarian cancer, particularly in patients with MGCT.

## Figures and Tables

**Table 1:** Fertility results after fertility-sparing surgery in series of women with borderline ovarian tumors and a desire to conceive.

	Fertility-Sparing Surgery (*n*)	Pregnancy Rate (%)
Boran et al. (2005) [16] Early Advanced	25 25 0	40
Fauvet et al. (2005) [17] Early Advanced	62 62 0	32
Park et al. (2009) [18] Early Advanced	184 181 3	73
Kanat-Pektas et al. (2011) [19]	55	52
Koskas et al. (2011) (*) [20]	31	38
Song et al. (2011) [21] Early Advanced	155 150 5	88
Lee et al. (2017) [22] Early Advanced	108 105 3	81
Delle Marchette et al. (2019) [23] Early Advanced	535 438 97	84
Lu et al. (2019) [24] Early Advanced	21 0 21	40
Candotti et al. (2020) [25] Early Advanced	85 74 11	73
Chevrot et al. (2020) [26] Early Advanced	52 30 22	63
Jia et al. (2020) [14] Early Advanced	79 43 36	47
Gouy et al. (2020) [12] Early Advanced	65 0 65	69
Plett et al. (2020) [27] Early Advanced	95 77 18	82.9

* Only patients with mucinous borderline ovarian tumors in this series. The observed pregnancy rates are between 32 and 88%.

**Table 2:** Literature review of recurrence risk and survival rates after fertility-sparing surgery for patients with epithelial ovarian cancer.

	Number of Patients *n*	Histologic Type	Stage IA *n*	Stage IB *n*	Stage IC *n*	Grade 1 *n*	Grade 2 *n*	Grade 3 *n*	Recurrence *n* (%)	Death *n* (%)	5 Year Recurrence Free Survival %
Zanetta et al. (1997) [51]	56	All types	32	2	22	35	14	7	5 (8.9)	3 (5.3)	
Schilder et al. (2002) [52]	52	All types	42	0	10	38	9	5	5 (9.6) S 2/10, M 2/25, E 1/10, CC 0/5	2 (3.8)	
Morice et al. (2005) [53]	34	All types	30	0	3	15	15	4	10 (29.4) S 2/3, M 5/21, E 1/5, CC 1/2	4 (11.7)	
Borgfeldt et al. (2007) [54]	11	All types	10	0	1	9	1	1	1 (9)	1 (9)	
Park et al.(2008) [55]	62	All types	36	2	21	48	5	9	11 (17.7) S 0/7, M 7/41, E 1/8, CC 2/4	6 (9.7)	
Anchezar et al. (2009) [56]	16	All types	11	0	5	14	1	1	2 (12.5)	1 (6.2)	
Schlaerth et al. (2009) [57]	20	All types	11	0	9	15	5	1	3 (15)	3 (15)	
Kwon et al. (2009) [58]	21	All types	17	0	4	16	3	2	1 (4.7)	0	
Wright et al. (2009) * [59]	432	All types	370	0	62	157	92	37	NA		94%
Satoh et al. (2010) [60]	211	All types	126	0	85	160	15	36	18 (8.5) S 3/27, M 6/126, E 4/27, CC 5/30	5 (2.4)	
Kajiyama et al. (2010) [61]	60	All types	30	1	29	41	7	12	8 (13.3)	7 (11.7)	
Hu et al. (2011) [62]	94	All types	46	8	28	64	13	1	2 (2.4)	NA	
Kajiyama et al. (2011) [63]	40	Mucinous	27	0	14	NA	NA	NA	NA	NA	97.5%
Cheng et al. (2012) [64]	17	All types	10	0	6	15	2	0	1 (5.9)	0	
Fruscio et al. (2013, 2016) [65,66]	240	All types	130	2	105	141	70	29	27 (11.2) S 11/62, M 8/99, E 6/60, CC 2/17	11 (4.6)	
Kashima et al. (2013) [67]	18	All types	0	0	18	14	0	4	5 (27.7)	4 (22.2)	
Kajiyama et al. (2014) [68]	94	All types	43	0	51	59	14	4	14	11	84.3%
Lee et al. (2015) [69]	35	Mucinous	21	0	13	27	5	1	6 (17.1)		91%
Ditto et al. (2015) [70]	70	All types	46	2	15	36	24	9	NA		98%
Bentivegna et al. * (2016) [71]	673	All types	396	46	231	442 **	126 **	58 **	79/673 (12) S 20/128 (16) M 30/344 (9) E 16/128 (12) CC 10/60 (17)		
Gouy et al. (2017) [72]	21	Mucinous	9	0	12	9	5	1	2 (9,5%)	0	90.5% (median follow: 46 months (*r* 1–169)
Jiang et al. (2017) [73]	52	All types	19		33	45	4	1	5 (9,6%)	NA	91%
Melamed et al. (2017) [74]	825	All types	546	0	279	298	201	111		30 (3.6)	
Ratanasrithong et al. (2018) [75]	28	All types				17	1	5	4 (14.3)	1 (3.6)	
Hanatani et al. (2019) [76]		All types	325								
Kajiyama et al. (2019) [77]	8 (with cystectomy)	All types	3	0	5	8	0	0	2 (25)	1 (12.5)	
Bogani et al. (2020) [78]	34	All types	21	2	9	28		6	7 (20.6)	2 (5.9)	
Liu et al. *** (2020) [79]	2223	All types									
Watanabe et al. (2020) [80]	29	All types	14	0	15	13	10	3	5 (17.2)	2 (6.9)	90.9 for IA/IC1 43.8 for IC3
Zhang et al. (2020) [81]	5	Mucinous	5	0	0				0	0	

* Review including data from [51,52,53,56,60,65,82]; ** stage 1A et 1C (stage IB excluded); *** meta analysis including [51,59,66,69,70,73,77]; S: Serous, M: Mucinous, E: Endometriod, CC: Clear cell; Recurrence rate by histologic type has been indicated for series including more than 10 patients with recurrence.

**Table 3:** Fertility outcomes after fertility-sparing surgery for patients treated for epithelial ovarian cancer.

Author (Year)	Number of Patients *n*	Number of Patients Wishing to Conceive *n* (%)	Number of Pregnant Patients *n* (%)	Live Births *n*
Schilder et al. (2002) [52]	52	24 (46%)	17 (71%)	26
Park et al. (2008) [55]	62	19 (30%)	15 (79%)	22
Schlaerth et al. (2009) [57]	20	15 (75%)	6 (40%)	9
Kwon et al. (2009) [58]	21	5 (24%)	5 (100%)	5
Satoh et al. (2010) [60]	211	84 (40%)	45 (53%)	56
Cheng et al. (2012) [64]	17	8 (47%)	5 (62%)	6
Fruscio et al. (2013, 2016) [65,66]	240	105 (44%)	84 (80%)	91
Kashima et al. (2013) [67]	18	10 (55%)	5 (50%)	7
Gouy et al. (2017) [72]	21	21 (100%)	4 (19%)	6
Jiang et al. (2017) [73]	52	34 (65%)	32 (94%)	28
Ratanasrithong et al. (2018) [75]	28	7 (25%)	4 (6%)	4
Kajiyama et al. (2019) [77]	8	7 (88%)	4 (6%)	4
Total	802	339/802 (42%)	226/339 (67%)	264

**Table 4:** Literature review of fertility outcomes after fertility-sparing surgery in germ cell tumors (series published after 1995).

Author	*N* pts	*N* Conservative	Menstruation Maintained	*N* Pregnancies	*N* Conservative II/III/IV
Peccatori et al. 1995 [88]	129	108	?	?	37
Mitchell et al. * 1999 [89]	69	50	24/26	11	?
Brewer et al. ** 1999 [90]	26	16	14	5	?
Perrin et al. 1999 [91]	45	45	During chemotherapy 50% became amenorrhoeic 96% resumed normal menstrual function on completion	7 healthy babies in the chemotherapy group	
Tewari et al. 2000 [92]	72	46	?	?	1 *****
Low et al. 2000 [93]	74	74	43/45	19/20	19
Zanetta et al. 2001 [94]	169	138	128/130	55 in 32 pts	46
Tangir et al. 2003 [95]	106	64	32/40 ***	38 in 29 pts	11 (9 pregnancies)
Zanagnolo et al. 2004 [96]	55	39	26	11	11
Boran et al. ** 2005 [97]	23	23	19/23 ***	6 in 5 pts	8 (4 pregnancies)
Ayhan et al. * 2005 [98]	29	15	10 ****	3	?
Kang et al. * 2008 [99]	20	15	15	2	?
De la Motte Rouge et al. * 2008 [100]	52	41	39/40	19 in 12 pts	4 pregnancies
Ertas et al. 2014 [101]	42	31	23/27 with chemotherapy	17 by 21 patients who attempted conception	13
Satoh et al. * 2015 [102]	211	157	109/109	29 in 40 pts	?
Yang et al. (2016) [103]	106	59	45	33 in 31 pts (39 desired a pregnancy)	
Ghalleb et al. (2017) [104]	1	1	1	3	1
Park et al. (2017) [105]	171	171	106/124 patients (85.5%) had regular menstruation, 12/124 patients (9.7%) had irregular menstruation, and 6/124 patients (4.8%) had premature menopause	15/20 patients (75%) succeeded in achieving 21 pregnancies, 13/20 of the patients (65%) gave birth to 20 healthy babies.	46
Ratanasrithong et al. (2018) [75]	22	22		4 in 4 patients (8 desired a pregnancy)	
Duhil (2018) et al. ** [106]	48	36		13 patients (36%) had 22 pregnancies resulting in 17 healthy born children	
Lakshmanan et al. (2018) [107]	39	14	9	1	
Tamauchi et al. (2018) [108]	105	105	57 (among 72 with chemotherapy, and NA = 13)	42 patients (45 desired a pregnancy) had 64 pregnancies resulting in 56 healthy born children (among 40 patients)	31
Dellino et al. (2020) [109]	28	24	Of 19 women with chemotherapy, 15 (78%) reported regular menstrual cycles during and after chemotherapy; the remaining 4 (21%) presented amenorrhea during chemotherapy but reported regular cycles after the end of treatment.	5 of 5 women who tried to get pregnant succeeded spontaneously.	5

* Papers reporting exclusively Endodermal Sinus Tumor or non-dysgerminomatous tumors; ** paper reporting only dysgerminomatous tumors; *** menstruation considered as “similar” to before chemotherapy; **** five patients excluded from menstruation assessment because of lethal recurrence; ***** pregnant patient. Pts: patients; ?: unknown data.
